# The Potential Roles of Glial Cells in the Neuropathogenesis of Cerebral Malaria

**DOI:** 10.3389/fcimb.2021.741370

**Published:** 2021-10-07

**Authors:** Nana Efua Andoh, Ben Adu Gyan

**Affiliations:** ^1^ Noguchi Memorial Institute for Medical Research, Department of Parasitology, University of Ghana, Accra, Ghana; ^2^ Noguchi Memorial Institute for Medical Research, Department of Immunology, University of Ghana, Accra, Ghana

**Keywords:** cerebral malaria, astrocytes, microglia, glial cells, *Plasmodium*, blood-brain barrier

## Abstract

Cerebral malaria (CM) is a severe neurological complication of malaria caused by the *Plasmodium falciparum* parasite. It is one of the leading causes of death in children under 5 years of age in Sub-Saharan Africa. CM is associated with blood-brain barrier disruption and long-term neurological sequelae in survivors of CM. Despite the vast amount of research on cerebral malaria, the cause of neurological sequelae observed in CM patients is poorly understood. In this article, the potential roles of glial cells, astrocytes, and microglia, in cerebral malaria pathogenesis are reviewed. The possible mechanisms by which glial cells contribute to neurological damage in CM patients are also examined.

## 1 Introduction

Malaria is a life-threatening disease with over 229 million cases and 409,000 deaths recorded worldwide in 2019. Most of these deaths occur among children under 5 years of age in Sub-Saharan Africa (World Health Organization, 2020). Malaria is caused by the protozoan parasite *Plasmodium* species (phylum Apicomplexa) of which *Plasmodium falciparum* (*P. falciparum*) is the deadliest of the species (reviewed by White, 2008).

Malaria is transmitted when an infected *Anopheles* mosquito deposits sporozoites into the dermis of the human host (Matsuoka et al., 2002; Amino et al., 2008). The sporozoites then travel to the liver where the sporozoites mature into schizonts within 5 to 16 days (Matsuoka et al., 2002). Subsequently, the schizonts rupture and release merozoites that can invade red blood cells (Dvorak et al., 1975; Weiss et al., 2015). The invading merozoites develop into immature trophozoites, then into schizonts. These mature schizonts burst and release merozoites into the bloodstream to invade uninfected red blood cells and start the cycle again (Dvorak et al., 1975). Some blood-stage merozoites form gametocytes that are taken by mosquitoes during a blood meal (Bruce et al., 1990; Buchholz et al., 2011). The blood-stage parasites (in all malaria species) are responsible for the clinical symptoms of malaria. Patients with uncomplicated malaria experience symptoms such as headaches, fever, chills, muscle aches, fatigue, nausea and vomiting (World Health Organization, 2020). In some patients with *P. falciparum* malaria, the disease may progress to severe malaria and patients may develop pathologies such as acute renal failure, liver and lung dysfunction, hypoglycaemia, severe anaemia, placental malaria and cerebral malaria (WHO, 2014).

## 2 Cerebral Malaria

Cerebral malaria (CM) is one of the most severe forms of *P*. *falciparum* infection and is associated with high death rates and long-term neurological sequelae in patients who survive CM. The World Health Organization (WHO) defines CM as a diffuse encephalopathy state. Diagnosis is typically given from a Glasgow Coma Score of < 11/15 for adults or a Blantyre Coma Scale of < 2 for children, an unarousable coma for at least an hour after a seizure, and/or detection of asexual forms of *P. falciparum* parasites in blood smears with the absence of factors that could cause a coma (e.g. hypoglycaemia or meningitis) (WHO, 2014, reviewed by Idro et al., 2005).

The clinical hallmark of CM is the presence of a coma with convulsions (WHO, 2014). With treatment, 15-20% of children still die from CM and long-term neurological with cognitive deficits are observed in approximately 25% of children who survive (WHO, 2014). Neurological deficits occur more often in children than in adults; it is hypothesized that children are more susceptible to neurological injury (reviewed by Hawkes et al., 2013). In adults, CM is part of a multi-organ disorder including renal failure and pulmonary oedema, symptoms that rarely occur in children (WHO, 2014).

The pathogenesis of cerebral malaria is multifaceted with sequestration, inflammation, and brain endothelial cell dysregulation all contributing to its aetiology. This review will focus on the role of glial cells in CM pathogenesis.

## 3 The Role of the Blood-Brain Barrier in Cerebral Malaria

### 3.1 The Blood-Brain Barrier

**Figure 1:**
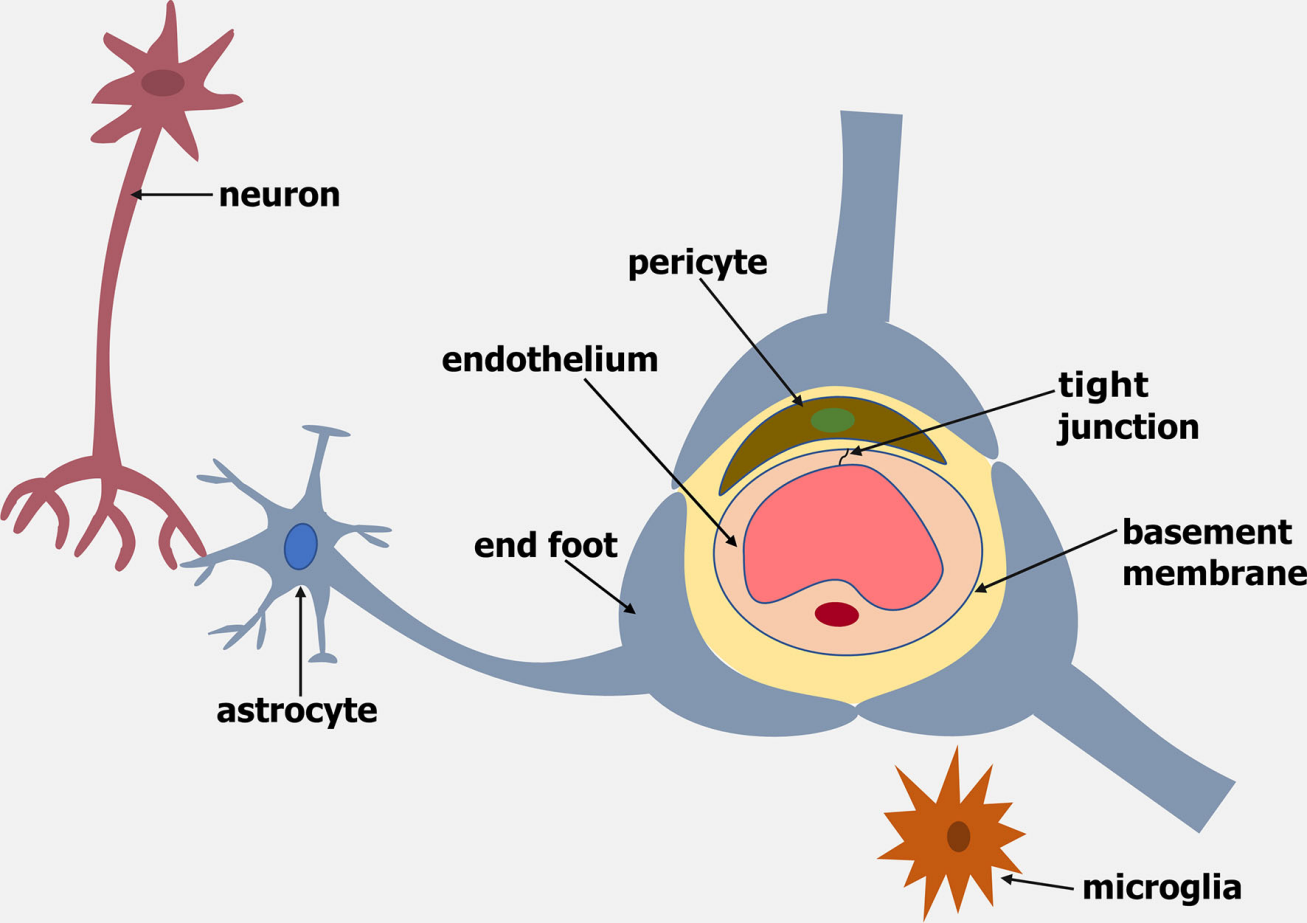
Schematic representation of the Neurovascular Unit (NVU). The NVU is made up of an endothelium, basement membrane, pericytes, microglia, astrocytes and neurons.

The blood-brain barrier (BBB) is a dynamic barrier formed by endothelial cells (ECs) that controls the movement of molecules, plasma proteins, pathogens and cells between the blood and brain. The brain endothelium of the BBB is part of a cellular complex known as the neurovascular unit (NVU), which is composed of a basement membrane, pericytes, astrocytes, microglia and neurons (reviewed by Abbott and Friedman, 2012) (**Figure 1**).

Brain ECs (BECs) vary from peripheral ECs in that they have no fenestrae, minimal pinocytic activity, a continuous basement membrane, a negatively charged luminal surface and the existence of tight junctions (reviewed by Daneman and Prat, 2015). Tight junctions and adherens junctions between BECs produce a strong BBB with a very high transendothelial electrical resistance (TEER) that is 50-100 times tighter than peripheral ECs (reviewed by Abbott et al., 2010).

### 3.2 Endothelial Cell Activation During Cerebral Malaria

**Figure 2:**
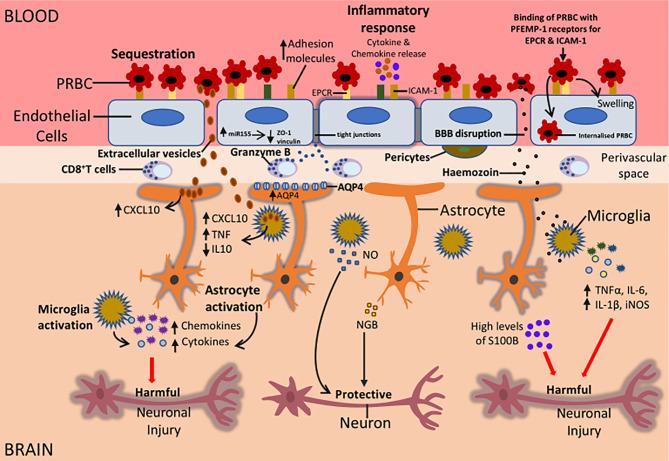
Potential mechanisms that could be responsible for the neurological sequelae observed in survivors of CM. During CM, sequestration of PRBC to the BBB can result in the activation of the endothelial cells of the BBB. This leads to an increased expression of adhesion molecules on endothelial cells, increased release of proinflammatory cytokines and chemokines, increased miR155 expression and internalisation of PRBC by endothelial cells. Also, CD8^+^ T cells in the perivascular space can release granzyme that can induce apoptosis of endothelial cells. All these factors can cause BBB disruption resulting in the movement of parasite-derived factors such as Hz, EVs, cytokines and chemokines into the brain causing activation of microglia and astrocytes. Increased expression of AQP4 can result in the influx of fluid causing swelling of astrocytes and this can result in oedema. Activation of glial cells can be beneficial or damaging depending on the type of injury. Activated glial cells can release cytokines and chemokines such as IL-1β, TNFα, CXCL10, CXCL9 that can impair neuronal function. This can result in long term neurological sequelae in CM survivors. On the other hand, activation of glial cells could protect neurons during CM. The release of NO by microglia and NGB by astrocytes can protect neurons from neuronal damage. Understanding the mechanisms that are involved in glial activation and neuronal damage during CM can lead to the development of adjunct therapies that can help alleviate the burden of neurological sequelae in patients who survive CM.

BECs play an active role in the pathogenesis of a number of central nervous system (CNS) disorders such as CM. In the late stages of the intraerythrocytic cycle, *Plasmodium falciparum* infected red blood cells (PRBC) expressing *Plasmodium falciparum* erythrocyte membrane protein-1 (PfEMP1) bind to several receptors including the intercellular adhesion molecule (ICAM-1) and endothelial protein C receptor (EPCR) on brain endothelial cells in a process known as sequestration (Brown et al., 2013; Lau et al., 2015; Avril et al., 2016) (**Figure 2**). Results from human post-mortem and *in vitro* studies suggest that sequestration is involved in CM pathogenesis. A high accumulation of PRBC in the cerebral vasculature was observed in the brains of patients who died from CM (MacPherson et al., 1985; Turner et al., 1994; Ponsford et al., 2012). Studies by Tripathi et al. (2006) showed a dose and time-dependent increase in ICAM-1 levels when PRBC were co-cultured with human brain endothelial cells (HBEC) (Tripathi et al., 2006).

Examination of post-mortem CM brain tissues of Vietnamese adults and Malawian children showed a reduction in junction proteins vinculin, zonula occludens-1 (ZO-1), and occludin in blood vessels where PRBC sequestration was present (Brown et al., 1999; Brown et al., 2001). *In vitro* coculture of PRBC from CM patients with human umbilical vein endothelial cells (HUVEC) also resulted in lower vinculin, ZO-1, and occludin levels (Susomboon et al., 2006). Similarly, a reduction in TEER was observed when PRBC were cocultured with HBEC (Tripathi et al., 2007). These findings indicated that sequestration of PRBC to BBB resulted in a reduction in junction proteins which implies loss of BBB integrity.

MicroRNA155 (miR155), a small noncoding molecule involved in neuroinflammation at the BBB, has also been shown to cause BBB disruption *in vitro*. Upregulation of miR155 induced by proinflammatory cytokines resulted in the reorganization of junction proteins and a 1.9 fold increase in HBEC permeability *in vitro* (Lopez-Ramirez et al., 2014). In ECM, genetic deletion of miR155 reduced endothelial cell activation and decreased BBB leak (Barker et al., 2017) Additionally, treatment with anti-miR155 decreased vascular leakage caused by serum from cerebral malaria patients close to basal levels in an *ex vivo* endothelial microvessel model (Barker et al., 2017). These results suggested that miR155 indirectly contributed to endothelial dysfunction and BBB disruption in CM (**Figure 2**).

Transmigration of PRBC into the brain endothelium can cause BBB disruption during CM (**Figure 2**). Studies by Adams et al. (2021) showed that incubation of PRBC expressing dual ICAM-1 and EPCR PfEMP-1 proteins, with HBEC, resulted in the internalization of PRBC by HBEC. This resulted in the swelling of the HBEC and BBB breakdown (Adams et al., 2021) (**Figure 2**). Also, *ex vivo* studies showed the presence of internalized PRBC within HBEC in post-mortem tissue samples from Indian patients who died from CM. This data indicates transmigration of PRBC into the brain endothelium during CM could be a contributing factor to CM pathogenesis (Adams et al., 2021).

Altogether these studies suggest that activation of the brain endothelium during CM can cause BBB disruption. This can lead to the movement of cytokines, chemokines and parasite-derived products into the brain parenchyma where they activate cells of the NVU such as astrocytes and microglia.

### 3.3 Astrocytes

Astrocytes are glial cells whose end-feet surround >99% of the brain capillaries (Mathiisen et al., 2010). They can be characterised into two broad morphologies: protoplasmic and fibrous astrocytes (Andriezen and Lond, 1893). Protoplasmic astrocytes are located in the grey matter whereas fibrous astrocytes can be found in the white matter (Zhang et al., 2019). Although astrocytes are broadly classified into these two groups, astrocyte heterogeneity can be observed within and between regions. Studies by Batiuk et al. (2020) using single-cell RNA sequencing and transcriptomics observed 5 different astrocyte populations in the hippocampus and cortex of adult mice (Batiuk et al., 2020). Astrocytes are involved in many key processes in the brain. They protect and support neurons by regulating synapse formation and maintaining brain homeostasis (reviewed by Dossi et al., 2018). Astrocytes also secrete factors that are important for the formation of a functional BBB (reviewed by Dossi et al., 2018). In response to CNS injury, astrocytes undergo a gradation of cellular, molecular, and functional changes known as astrogliosis (reviewed by Dossi et al., 2018). Astrogliosis is characterised by an increase in glial fibrillary acidic protein (GFAP) and can range from mild to moderate or severe alterations in astrocytes where compact scar formations occur (referred to as glial scar) (reviewed by Dossi et al., 2018). Astrogliosis is thought to initially repair and limit the level of damage during CNS injury, however, it inhibits regeneration and later causes detrimental effects in CNS disorders.

Reactive astrocytes have recently been grouped into two different types in the adult CNS, A1 and A2. Studies by Liddelow et al. (2017) showed that A1 type astrocytes were induced by activated microglia *via* secretion of interleukin-1 alpha (IL-1α), tumour necrosis factor (TNF) and C1q. A1 astrocytes *in vivo* and *in vitro* released an unknown neurotoxin that caused apoptosis of neurons and oligodendrocytes (Liddelow et al., 2017). However, A2 astrocytes were induced by ischaemia and upregulated neurotrophic genes that promoted neuronal survival (Liddelow et al., 2017). These results indicate that astrocytes can play a neuroprotective or detrimental role in the brain during different neurological disorders. Astrocytes are heterogeneous and difficult to study, thus it is possible that more subtypes of reactive astrocytes exist. Reactive astrocytes may also possess unique cellular and molecular features that only occur in specific neuropathology. There is still a lot of research that needs to be done to understand the mechanisms and pathways that are involved in the induction of reactive astrocytes in CNS disorders.

### 3.4 Microglia

Microglia are the resident immune cells of the CNS and play a vital part in the immune response (Thurgur and Pinteaux, 2019). They are restricted to the brain and self-renew throughout life without the involvement of circulating blood cells (reviewed by Arcuri et al., 2017).

Microglia are very heterogeneous, differing in population densities across brain regions, and more microglia are present in the gray matter than the white matter. White matter microglia have elongated somata aligned parallel to fibres whilst microglia in circumventricular organs have a compact morphology and microglia in the grey matter are radially ramified (reviewed by Arcuri et al., 2017). Microglia cells express the pattern recognition receptors (PRRs) that survey their microenvironment and recognise indicators for injury known as pathogen-associated molecular pattern molecules (PAMPs) and damage-associated molecular patterns (DAMPs) (reviewed by Arcuri et al., 2017). Upon acute brain injury microglia transition from a surveillance mode to an activated state where they undergo significant morphological changes, decrease the number of processes and release pro-and anti-inflammatory cytokines (reviewed by Arcuri et al., 2017).

Previously, microglia were classified into two different activation phenotypes. The M1 (classical activation) phenotype referred to a proinflammation state in which microglia release mediators TNFα, IL-1β, IL-6, reactive oxygen species (ROS) and nitric oxide (NO); whereas the M2 (alternative activation) phenotype referred to an anti-inflammatory state where microglia released trophic factors such as IL-4, IL-10 and transforming growth factor beta (TGFβ) (reviewed by Figarella et al., 2020). However, this classification did not reflect the heterogeneity of the microglia phenotype and now it is known that microglia can assume a broad spectrum of different activation profiles. This heterogeneity of the microglial phenotype depends on the anatomical region of microglia and their close interactions with cells such as microglia, astrocytes, neurons and oligodendrocytes (reviewed by Figarella et al., 2020). Initially, activated microglia were thought to be only detrimental to the CNS, however, several studies suggest that activated microglia have both detrimental and beneficial functions. Microglia can also be neuroprotective by producing factors such as brain-derived neurotrophic factor, glial cell-derived neurotrophic factor, and nerve growth factor, that can help prevent neuronal damage (reviewed by Fakhoury, 2018).

## 4 Astrocyte and Microglia Activation During Cerebral Malaria

Astrogliosis and activation of microglia have been observed in human cerebral malaria (HCM) and experimental CM (ECM). In the brain of Vietnamese patients who died from severe malaria, an increase in astrogliosis and fragmentation of astrocyte processes were observed (Medana et al., 2002). Similarly, mild to moderate astrogliosis was shown in the brain tissues of Malawian children who had died from CM (Dorovini-Zis et al., 2011). Accumulations of microglia around small veins were observed in the brain parenchyma of English travellers who died from cerebral malaria (Janota and Doshi, 1979). Post-mortem studies showed that markers of early microglia activation, MRP8, and MRP14, were widely expressed by microglia in the white and grey matter, and in blood vessels containing sequestered parasites during CM (Schluesener et al., 1998). Although these studies showed that astrocytes and microglia were being activated in CM, they failed to discuss the impact of glial activation on the brain during CM. This could largely be due to limited accessibility to human post-mortem brain tissues of CM patients. Due to these restrictions, most of the information on the role of glial cells in CM pathogenesis has been performed in mouse models of CM where C57BL/6 or CBA mice are infected with the malaria parasite *P. berghei* ANKA (PbA) to develop ECM (reviewed by Idro et al., 2010).

The murine model of CM supports a role for glia activation in the CM pathogenesis. Transcriptomic analysis showed proliferation of microglia prior to the onset of ECM (Capuccini et al., 2016; Talavera-López et al., 2018). Astrocyte and microglia activation were also shown to occur before the onset of ECM and neurological symptoms in the fatal murine model of ECM (Medana et al., 1996; Medana et al., 1997). As ECM progressed, morphological changes such as retraction of ramified processes, large soma, amoeboid appearance, and extensive vacuolation occurred in microglia (Medana et al., 1997). At the terminal stage of this disease when mice were displaying neurological symptoms, loss of astrocyte processes contacting retinal vessels were observed and this could have been caused by the immune response elicited by PbA (Medana et al., 1996; Medana et al., 2001). Due to the crucial roles astrocytes play in maintaining brain homeostasis and protecting neurons, damage to astrocytes as observed in these studies can have detrimental effects on neuronal functions. Thus, the studies above show that glial cells may be a key player in the neuropathogenesis of CM.

### 4.1 Neuroinflammatory Markers of Glial Cells During CM

Neuroinflammation is a common characteristic of most CNS disorders and insults. It is associated with activation of astrocytes and microglia with marked production of cytokines, chemokines, proinflammatory mediators including C-X-C motif chemokine ligand 10 (CXCL10), and TGFβ, and BBB disruption in neurological diseases such as CM (**Figure 2**).

The chemokine CXCL10 also known as interferon-γ inducible protein-10 (IP-10) is constitutively expressed in astrocytes, microglia, and neurons (reviewed by Jiang et al., 2017) and markedly increased in reactive astrocytes in CNS disorders such as Alzheimer’s disease (AD) (Xia et al., 2000). CXCL10 was significantly elevated in the serum and CSF of Ghanaian children with CM (Armah et al., 2007) suggesting that CXCL10 played a role in neuroinflammation observed during CM. Indeed, *in vivo*, upregulation of genes involved in chemokine production of CXCL9, CXCL10, CCL8 and CCL12 was observed in the microglia of mice infected with PbA using genome wide transcriptomic analysis (Capuccini et al., 2016). CXCR3, the receptor of CXCL10 is known to play a key role in the recruitment of T cells into the brain. Studies by Campanella et al. (2008) showed CD8^+^ T cell infiltration into the brain of CXCR3 knock-out mice infected with PbA was significantly reduced by 300% compared with wild-type mice (Campanella et al., 2008). Data from both studies suggested that upregulation of CXCL10 by activated glial cells could induce the recruitment of CD8^+^ T cells into the brain by binding to CXCR3 highly expressed on the surface of CD8^+^ T cells during ECM.

CD8^+^ T cells were previous shown to only exist in ECM, however, recent studies confirmed the presence of CD8^+^ T cells in the brains of Malawian children who died from CM (Barrera et al., 2019; Riggle et al., 2020). In these studies, CD8^+^ T cells were found in the intravascular and perivascular space of the brain but were absent from the brain parenchyma (Barrera et al., 2019; Riggle et al., 2020). CD8^+^ T cells were also observed in the leptomeninges and choroid plexus in HCM samples suggesting possible routes of entry into the superficial areas of the brain (Barrera et al., 2019). Also, granzyme B (which is a protease known to mediate cellular apoptosis) was found to be expressed by CD8^+^ T cells that were in contact with endothelial cells (Riggle et al., 2020). Results from these studies suggest that CD8^+^ T cells do not enter the brain parenchyma during HCM and thus may not have a direct influence on the neuroinflammation caused by microglia and astrocytes during HCM. However, astrocytes can extend their processes across the perivascular space in the brain, bringing them in contact with activated CD8^+^ T cells that can then target astrocyte processes by releasing granzyme B (**Figure 2**). This could result in astrocyte activation and the release of chemokines and cytokines leading to further neuroinflammation in the brain during HCM (**Figure 2**).

TGF-β is another cytokine that has been shown to be involved in the neuropathogenesis of CM TGF-β expression was upregulated in the brain sections of CM patients in an intravascular and perivascular distribution but not in an intraparenchymal distribution (Armah et al., 2005). In the post-mortem brain tissues of CM patients, a significant increase in TGF-β-1 expressing astrocytes was observed around the brain vessels with malaria pigment. Also, TGF-β2 expressing microglia within Dürck granulomas and ring haemorrhages, and TGF-β3 expressing endothelial cells around the brain vessels were observed in the post-mortem brain tissue of CM patients (Deininger et al., 2000). These results suggest that TGF-β expressed by glial cells contributes to neuroinflammation during CM. However, the exact role of TGF-β expressed by glial cells during CM has not been explored. In other neurological disorders, TGF-β expression by glial cells can be beneficial or detrimental depending on the disease. Overproduction of TGF-β1 in astrocytes accelerated disease progression and reduced microglia function in amyotrophic lateral sclerosis (ALS) mice (Endo et al., 2015). On the contrary, TGFβ-1 derived from astrocytes protected synapses against amyloid B oligomers (the main component of amyloid plaque in AD) (Diniz et al., 2012). Also, TGF-β treatment after intracerebral haemorrhage resulted in a reduction in microglia inflammation and an increase in functional recovery *in vivo* (Taylor et al., 2016). Reduced levels of TGF-β in malaria patients were associated with disease severity. TGF-β1 levels in the serum and plasma of cerebral malaria patients were significantly reduced compared to uncomplicated malaria patients (Chaiyaroj et al., 2004; Hanisch et al., 2015).

High levels of TGF-β are associated with anti-inflammatory effects whereas low levels of TGF-β are associated with proinflammatory effects. Thus, it is possible that during CM, the amount of TGF-β expressed by glial cells determines the severity of CM. Increased levels of TGF-β could suppress neuroinflammation in CM and low levels of TGF-β could exacerbate neuroinflammation in CM. Further studies needed to be done to determine the exact role of TGF-β in the neuropathogenesis of CM.

### 4.2 Dysregulation of Coagulation During CM

Coagulation factors have also been shown to be important players in inflammation in several neurological diseases including CM (**Figure 2**). Indeed, dysregulation of coagulation has been found in both HCM and murine CM. In the post-mortem brain tissues of CM patients, sequestration was associated with microvascular thrombi and perivascular haemorrhages (Dorovini-Zis et al., 2011).

*In vivo*, vascular thrombi containing adherent leukocytes were observed in IL-10 knockout (IL-10 KO) mice that had been infected with the rodent malaria parasite *P. chabaudi*. In this study, astrocytes and microglia were also detected in the brain parenchyma clustered near vessels with thrombi (Wilson et al., 2018). Interestingly, neutralisation of TNF and coagulation caused a marked reduction in intravascular thrombi and decreased astrocyte and microglial activation in *P. chabaudi* IL-10 KO mice (Wilson et al., 2018). These results could indicate that leukocytes contribute to intravascular coagulation during malaria and also suggest that there is an association between inflammation, coagulation, and glial activation during murine cerebral malaria (Wilson et al., 2018). This study suggests that inflammatory leukocytes localised in thrombi could produce cytokines that can cross the BBB and activate glial cells during cerebral malaria thereby contributing to neuroinflammation in the brain parenchyma during cerebral malaria.

### 4.3 Extracellular Vesicles and Glial Activation During CM

In addition to proinflammatory cytokines, extracellular vesicles (EVs) have also been shown to contribute to inflammation during CM (**Figure 2**). Extracellular vesicles (EVs) are produced from different subcellular compartments and are released into the extracellular space where they can influence cells within the vasculature such as endothelial cells. Studies by Combes et al. (2004) showed significantly higher levels of endothelial EVs were present in the plasma of Malawian children suffering from CM compared to patients with severe and uncomplicated malaria (Combes et al., 2004). Elevated levels of platelet, erythrocytic, endothelial, and leukocyte-derived EVs were observed in patients with *P. falciparum* malaria that had neurological dysfunctions (Pankoui Mfonkeu et al., 2010).

ATP-binding cassette transporter (ABCA) 1 knockout (KO) mice, that had a reduced ability to produce EVs, were protected against ECM and did not display cerebral symptoms (Combes et al., 2005). In addition, reduced inflammation accompanied by a notable decrease in TNFα was observed in PbA infected ABCA1 KO mice compared to wild-type mice (Combes et al., 2005). Data from the studies above indicate that EVs promote inflammation in CM and ECM and could contribute to the neurological syndrome observed in severe malaria.

EVs derived from PRBC and PbA infected red blood cells (PbARBC) have also been shown to interact with cells in the NVU such as astrocytes and microglia. *In vitro*, coculturing of PbARBC cells with a mixed astrocyte and microglia culture resulted in an uptake of EVs by astrocytes and phagocytosis of PbARBC by microglia (Shrivastava et al., 2017). Subsequently, this caused an increase in the CXCL10/IFN inducible protein 10 (IP10) secretion (Shrivastava et al., 2017). This indicated that the internalisation of EVs from malaria-infected red blood cells extracellular vesicles (MiREVs) by glial cells could contribute to neuroinflammation observed during CM. Recent studies by Mbagwu et al. (2020) showed the uptake of MiREVs by microglia generated from human blood monocytes in the perinuclear region led to morphological changes in microglia such as retraction of processes and swelling of the cell body suggesting microglial activation (Mbagwu et al., 2020). Furthermore, treatment of microglia with MiREVs derived from supernatants from *P. falciparum* culture caused an upregulation in the gene expression of TNFα and the downregulation in the gene expression of the anti-inflammatory cytokine IL10 (Mbagwu et al., 2020). This suggested that MiREVs contributed to inflammation in CM by inducing upregulation of the inflammatory cytokine TNFα and decreasing the expression of the immune-suppressive cytokine IL-10 in microglia.

Data from the above studies suggest that MiREVs may interact with glial cells during CM and this could exacerbate neuroinflammation in CM.

### 4.4 Role of Parasite-Derived Products on Glial Activation During CM

Malaria derived products such as haemozoin (Hz) and heme oxygenase-1 (HO-1) also contribute to neuroinflammation in CM (**Figure 2**). During the intraerythrocytic stages of malaria, digestion of haemoglobin by the *Plasmodium* parasite produces heme and this is stored in the parasite’s digestive vacuole as (Hz), a non-toxic crystalline polymer. Heme oxygenase-1 (HO-1) also degrades heme into carbon monoxide (CO), biliverdin, and iron (Schluesener et al., 2001). High levels of Hz correlate with disease severity in malaria patients (Lyke et al., 2003).

In the post-mortem brain tissues of CM patients, a significantly higher accumulation of Hz and platelets were observed, compared to the post-mortem brain tissues of severe malaria with anaemia patients and nonmalaria encephalopathy patients (Grau et al., 2003). The effect of Hz on cells of the NVU has recently been shown *in vitro* and *in vivo*.

A dose and time-dependent uptake of synthetic Hz (sHz) by astrocytes and neurons were observed *in vitro*, leading to apoptosis in both cells (Eugenin et al., 2019). This indicated that Hz was toxic to astrocytes and neurons and possibly contributed to the neurological sequelae observed in survivors of CM (Eugenin et al., 2019). Exposure of microglia to sHz caused a significant increase in TNFα, IL-6, IL-1β, inducible nitric oxide synthase (iNOS) mediated NO production expression and NLRP3 inflammasome activation *in vitro* (Velagapudi et al., 2019). However, treatment of mouse peritoneal macrophages (PM) with sHz following stimulation with lipopolysaccharide (LPS) impaired their ability to produce NO and TNFα but caused an increase in HO-1 (Taramelli et al., 1995; Taramelli et al., 2000). Interestingly, this effect was not observed in microglia treated with sHz where a reduction in NO and TNF α production was not observed. It was suggested that sHz induced oxidative stress in PM and this, in turn, inhibited the production of inflammation cytokines (Taramelli et al., 1995; Taramelli et al., 2000). HO-1 could also have suppressed iNOS production impairing NO synthesis in PM. Microglia are known to contain high amounts of antioxidant molecules and thus seem to be protected against oxidative stress (Vilhardt et al., 2017). This could be the reason sHz had no effect on microglia. Altogether results from these studies indicate that sHz could play a key role in neuroinflammation through activation of astrocytes and microglia and could contribute to the neurological damage observed in survivors of CM (**Figure 2**).

### 4.5 Aquaporin 4: A Player in Oedema During CM

The water protein channel Aquaporin 4 (AQP4) found in the astrocyte endfeet has been suggested to contribute to the neuropathogenesis of CM (**Figure 2**). AQP4 plays a key part in the control of water movement into and out of the brain and has been associated with vasogenic oedema and cytotoxic oedema in CM (Mohanty et al., 2017; Huang et al., 2019).

It remains unclear whether AQP4 plays a protective or deleterious role in CM. High levels of AQP4 were found to be expressed in areas of retinal whitening in CM patients due to retinal disruption (Barrera et al., 2018). Post-mortem studies showed increased expression of AQP4 in the brain stem of CM patients (Medana et al., 2011). In ECM, AQP4 was significantly higher in PbA infected mice susceptible to neurological manifestations such as palsies, convulsions, or ataxia (Ampawong et al., 2011). Thus, it was proposed that AQP4 played a detrimental role in ECM, and above a certain AQP4 expression threshold, neurovascular pathology occurred in mice (Ampawong et al., 2011). On the other hand, a reduced expression of AQP4 and a higher degree of oedema was observed in AQP4 knockout mice with ECM suggesting that AQP4 protected mice from oedema in ECM (Promeneur et al., 2013).

It is possible that the time point of injury and the type of oedema present in CM could determine whether AQP4 is beneficial or detrimental in cerebral malaria. Indeed, *in vivo* studies have shown AQP4 improves vasogenic oedema in the initial stages of spinal cord contusion through the reabsorption of water (Huang et al., 2019). At the middle stages of SCI, AQP4 facilitates the development of cytotoxic oedema, and inhibition of AQP4 formation reduces cytotoxic oedema and alleviates motor functions *in vivo* (Huang et al., 2019). Further studies need to be done to determine the exact role of AQP4 at the initial stage of CM and as the disease progresses.

### 4.6 S100B Proteins in CM

S-100B, a marker of astrocyte brain injury is associated with an increased risk of seizures during CM (**Figure 2**). In severe *falciparum* malaria, high levels of the astrocyte marker S-100B in the cerebrospinal fluid (CSF) of Kenyan children and Vietnamese adults were linked with an increased risk of recurrent seizures (Medana et al., 2007). Similarly, serum S-100B levels were markedly elevated in children with temporal lobe epilepsy, a condition characterised by recurrent seizures, compared to healthy controls (Calik et al., 2013). Results from both studies indicated that astrocytes contributed to seizures that occurred in CM and temporal lobe epilepsy. S-100B has also been suggested to contribute to neuroinflammation that occurs in CM. Treatment of co-cultures of astrocytes and neurons with S-100B resulted in apoptosis of neurons and this was dependent on NO produced from astrocytes (Hu et al., 1997). Also, S-100B induced IL-1β expression in microglia *in vitro* (Liu et al., 2005; Bianchi et al., 2011). Since S-100B is derived from astrocytes, these results indicate that astrocytes can coordinate certain elements of neuroinflammation caused by neurons and microglia. Thus, it is possible that during CM, S100B contributes to neuroinflammation in the brain by targeting microglia and neurons. However, the use of S-100B in the detection of brain damage is controversial and several factors affect the accuracy of S-100B as a biomarker for brain injury. S-100B has a short serum half-life, differs with age in children and extracerebral sources such as epithelial cells and adipose may contribute to S-100B serum levels (Ingebrigtsen et al., 1995; Woertgen et al., 2002; Gazzolo et al., 2003; Thelin et al., 2016; Bai et al., 2018). Hence, this raises questions concerning the reliability of S100B as a marker for brain injury. Thus, in future studies, the influence of age, sampling time and S-100B kinetics on S-100B levels should be considered when using S-100B as a biomarker for CNS damage in CM.

### 4.7 The Protective Role of Neuroglobin During CM

Neuroglobin (NGB) is a globin protein expressed in neurons and astrocytes. In neurons, neuroglobin has been shown to play a protective role in different pathologies such as AD and TBI by eliminating ROS. NGB expressed by astrocytes has also been detected under pathophysiological conditions in astrocytes.

Studies by DellaValle et al. (2010) showed that NGB was expressed by reactive astrocytes in murine models of cerebral malaria, TBI and autoimmune encephalitis (model of multiple sclerosis) but absent in the brain of healthy control mice (DellaValle et al., 2010). NGB was only observed in regions of the brain with severe pathology and astroglial scar, and BBB leakage was observed in all models with NGB astrocytes (DellaValle et al., 2010). Thus, it is possible that during ECM, following BBB damage, reactive astrocytes express and release NGB that might contribute to neuronal protection (**Figure 2**). However, the exact role of NGB expressed by astrocytes in CM has not been explored and further studies need to be done to understand the function of NGB secreted by astrocytes during CM. Altogether the studies in this review indicate that astrocytes and microglia contribute to the neuropathogenesis of cerebral malaria. Cross talk occurs between microglia and astrocytes through the secretion of molecules such as cytokines, chemokines, growth factors, NO and ROS (reviewed by Matejuk and Ransohoff, 2020).

Normal astrocyte-microglia cross-talk in disease is essential to support neuronal survival and function after acute injury whereas abnormal astrocyte-microglia cross-talk may promote neuroinflammation and neurological damage (reviewed by Matejuk and Ransohoff, 2020). Glial cells are heterogeneous, thus the impact of astrogliosis and microglia activation in the brain during CM will not be an all-or-nothing phenomenon but will occur in a context-dependent manner and this is regulated by specific signaling cascades associated with specific insults from the environment. The glial phenotype during CM could depend on the severity of disease, source of injury and region of the brain CM is occurring in.

## 5 Neurological Damage During Cerebral Malaria

Although the PRBC does not cross the BBB and remains in the lumen of the blood vessels of the brain, 25% of patients who survive CM are left with long term neurological sequelae such as speech and language impairment, cortical blindness, epilepsy and behavioural disorders such as attention deficit hyperactivity disorder (ADHD) (reviewed by Idro et al., 2016). These neurological sequelae in CM patients could be a result of neurological damage.

Proteins involved in neurological diseases such as AD have been observed in CM and ECM.

Studies by Delahaye et al. (2007) detected β-amyloid (Aβ) and a marked upregulation of the amyloid β (A4) precursor protein-binding family B in the brains of mice infected with PbA (Delahaye et al., 2007). Also, in the post-mortem brain tissues of adults who died from CM an upregulation of the β amyloid precursor protein (APP) was found (Medana et al., 2002). APP is cleaved by β and γ secretase to generate Aβ which contributes to neuronal dysfunction in AD (Steiner et al., 2018). Thus, the detection of APP and Aβ in the brain indicates neurological damage occurs during CM and ECM. Indeed, axonal and myelin injury linked with haemorrhages were observed in the white matter and brain stem of Malawian children who died from CM (Dorovini-Zis et al., 2011). Also, increased levels of axonal injury were observed in the post-mortem brain tissue of Vietnamese adults who died from CM (Medana et al., 2002). Elevated levels of Tau, an important biomarker for brain injury, was observed in the CSF of children with CM (Medana et al., 2007). Interestingly, increased levels of Tau in the CSF of CM patients were associated with long-term neurological impairment in children recovering from CM years after discharge (Datta et al., 2020). Altogether, results from these studies suggest that axonal injury during cerebral malaria could be the main contributor to neurological impairment that occurs in CM (**Figure 2**).

## 6 Conclusion

Cerebral malaria can cause long-term neurological damage in survivors of CM. The role of glial cells and neurons in HCM pathogenesis and neurological damage in HCM survivors is sparse and more research needs to be done in this area. This research should focus on understanding how the *Plasmodium falciparum* parasite interacts with components of the BBB such as endothelial cells, glial cells, pericytes and neurons to cause neurological damage during CM. This could lead to the development of adjunct therapies that can help alleviate the burden of neurological sequelae in patients who survive CM.

## Author Contributions

NA contributed to conception of the review. NA wrote the original draft of the manuscript. BG and NA contributed to the critical revision of the manuscript. All authors contributed to the article and approved the submitted version.

## Conflict of Interest

The authors declare that the research was conducted in the absence of any commercial or financial relationships that could be construed as a potential conflict of interest.

## Publisher’s Note

All claims expressed in this article are solely those of the authors and do not necessarily represent those of their affiliated organizations, or those of the publisher, the editors and the reviewers. Any product that may be evaluated in this article, or claim that may be made by its manufacturer, is not guaranteed or endorsed by the publisher.

## References

[B1] Abbott N. J. Friedman A. (2012). Overview and Introduction: The Blood-Brain Barrier in Health and Disease. Epilepsia 53, 1–6. doi: 10.1111/j.1528-1167.2012.03696.x PMC3625728 23134489

[B2] Abbott N. J. Patabendige A. A. Dolman D. E. Yusof S. R. Begley D. J. (2010). Structure and Function of the Blood-Brain Barrier. Neurobiol. Dis. 37, 13–25. doi: 10.1016/j.nbd.2009.07.030 19664713

[B3] Adams Y. Olsen R. W. Bengtsson A. Dalgaard N. Zdioruk M. Satpathi S.. (2021). Plasmodium Falciparum Erythrocyte Membrane Protein 1 Variants Induce Cell Swelling and Disrupt the Blood–Brain Barrier in Cerebral Malaria. J. Exp. Med. 218, e20201266. doi: 10.1084/jem.20201266 33492344 PMC7833209

[B4] Amino R. Giovannini D. Thiberge S. Gueirard P. Boisson B. Dubremetz J.. (2008). Host Cell Traversal Is Important for Progression of the Malaria Parasite Through the Dermis to the Liver. Cell Host Microbe 3, 88–96. doi: 10.1016/j.chom.2007.12.007 18312843

[B5] Ampawong S. Combes V. Hunt N. H. Radford J. Chan-Ling T. Pongponratn E.. (2011). Quantitation of Brain Edema and Localisation of Aquaporin 4 Expression in Relation to Susceptibility to Experimental Cerebral Malaria. Int. J. Clin. Exp. Pathol 5, 566–74. PMC3160608 21904632

[B6] Andriezen L. Lond M. (1893). The Neurologlia Elements in the Human Brain. BRAIN. Britain Med. J. 2, 227–230. doi: 10.1136/bmj.2.1700.227 PMC2422013 20754383

[B7] Arcuri C. Mecca C. Bianchi R. Giambanco I. Donato R. (2017). The Pathophysiological Role of Microglia in Dynamic Surveillance, Phagocytosis and Structural Remodeling of the Developing CNS. Front. Mol. Neurosci. 10, 191. doi: 10.3389/fnmol.2017.00191 28674485 PMC5474494

[B8] Armah H. B. Wilson N. O. Sarfo B. Y. Powell M. D. Bond V. C. Anderson W.. (2007). Cerebrospinal Fluid and Serum Biomarkers of Cerebral Malaria Mortality in Ghanaian Children. Malaria J. 6, 147. doi: 10.1186/1475-2875-6-147 PMC2186349 17997848

[B9] Armah H. Wiredu E. K. Dodoo A. K. Adjei A. A. Tettey Y. Gyasi R. (2005). Cytokines and Adhesion Molecules Expression in the Brain in Human Cerebral Malaria. Int. J. Environ. Res. Public Health Int. J. Environ. Res. Public Health 2, 123–131. doi: 10.3390/ijerph2005010123 16705810 PMC3814706

[B10] Avril M. Bernabeu M. Benjamin M. Brazier A. J. Smith J. D. (2016). Interaction Between Endothelial Protein C Receptor and Intercellular Adhesion Molecule 1 to Mediate Binding of Plasmodium Falciparum-Infected Erythrocytes to Endothelial Cells. mBio 7, e00615–e00616. doi: 10.1128/mBio.00615-16 27406562 PMC4958245

[B11] Bai M. Cai X. Li Z. Huang L. Gu C. Li Y.. (2018). Clinical Value of Serum S100B in Children With Epilepsy. Int. J. Clin. Exp. Med. 11, 13535–13540.

[B12] Barker K. R. Lu Z. Kim H. Zheng Y. Chen J. Conroy A. L.. (2017). miR-155 Modifies Inflammation, Endothelial Activation and Blood-Brain Barrier Dysfunction in Cerebral Malaria. Mol. Med. 23, 24–33. doi: 10.2119/molmed.2016.00139 28182191 PMC5364112

[B13] Barrera V. Haley M. J. Strangward P. Attree E. Kamiza S. Seydel K. B.. (2019). Comparison of CD8+ T Cell Accumulation in the Brain During Human and Murine Cerebral Malaria. Front. Immunol. 10, 1747. doi: 10.3389/fimmu.2019.01747 31396236 PMC6668485

[B14] Barrera V. MacCormick I. J. C. Czanner G. Hiscott P. S. White V. A. Craig A. G.. (2018). Neurovascular Sequestration in Paediatric P. Falciparum Malaria Is Visible Clinically in the Retina. eLife 7, e32208. doi: 10.7554/eLife.32208 29578406 PMC5898913

[B15] Batiuk M. Y. Martirosyan A. Wahis J. De Vin F. Marneffe C. Kusserow C.. (2020). Identification of Region-Specific Astrocyte Subtypes at Single Cell Resolution. Nat. Commun. 11, 1220. doi: 10.1038/s41467-019-14198-8 32139688 PMC7058027

[B16] Bianchi R. Kastrisianaki E. Giambanco I. Donato R. (2011). S100B Protein Stimulates Microglia Migration via RAGE-Dependent Up-Regulation of Chemokine Expression and Release. J. Biol. Chem. 286, 7214–7226. doi: 10.1074/jbc.M110.169342 21209080 PMC3044978

[B17] Brown H. Hien T. T. Day N. Mai N. T. H. Chuong L. V. Chau T. T. H.. (1999). Evidence of Blood-Brain Barrier Dysfunction in Human Cerebral Malaria. Neuropathol. Appl. Neurobiol. 25, 331–340. doi: 10.1046/j.1365-2990.1999.00188.x 10476050

[B18] Brown H. Rogerson S. Taylor T. Tembo M. Mwenechanya J. Molyneux M.. (2001). Blood-Brain Barrier Function in Cerebral Malaria in Malawian Children. Am. J. Trop. Med. Hyg. 64, 207–213. doi: 10.4269/ajtmh.2001.64.207 11442219

[B19] Brown A. Turner L. Christoffersen S. Andrews K. A. Szestak T. Zhao Y.. (2013). Molecular Architecture of a Complex Between an Adhesion Protein From the Malaria Parasite and Intracellular Adhesion Molecule 1. J. Biol. Chem. 288, 5992–6003. doi: 10.1074/jbc.M112.416347 23297413 PMC3581401

[B20] Bruce M. Alano P. Duthie S. Carter R. (1990). Commitment of the Malaria Parasite Plasmodium Falciparum to Sexual and Asexual Development. Parasitology 100, 191–200. doi: 10.1017/S0031182000061199 2189114

[B21] Buchholz K. Burke T. Williamson K. Wiegand R. Wirth D. Marti M. (2011). A High-Throughput Screen Targeting Malaria Transmission Stages Opens New Avenues for Drug Development. J. Infect. Dis. 203, 1445–1553. doi: 10.1093/infdis/jir037 21502082 PMC3080890

[B22] Calik M. Abuhandan M. Sonmezler A. Kandemir H. Oz I. Taskin A.. (2013). Elevated Serum S-100B Levels in Children With Temporal Lobe Epilepsy. Seizure 22, 99–102. doi: 10.1016/j.seizure.2012.10.012 23146618

[B23] Campanella G. S. V. Tager A. M. El Khoury J. K. Thomas S. Y. Abrazinski T. A. Manice L. A.. (2008). Chemokine Receptor CXCR3 and Its Ligands CXCL9 and CXCL10 Are Required for the Development of Murine Cerebral Malaria. Proc. Natl. Acad. Sci. 105, 4814–4819. doi: 10.1073/pnas.0801544105 18347328 PMC2290783

[B24] Capuccini B. Lin J. Talavera-López C. Khan S. M. Sodenkamp J. Spaccapelo R.. (2016). Transcriptomic Profiling of Microglia Reveals Signatures of Cell Activation and Immune Response, During Experimental Cerebral Malaria. Sci. Rep. 6, 39258. doi: 10.1038/srep39258 27991544 PMC5171943

[B25] Chaiyaroj S. Rutta A. Muenthaisong K. Watkins P. Na Ubol M. Looareesuwan S. (2004). Reduced Levels of Transforming Growth Factor-Beta1, Interleukin-12 and Increased Migration Inhibitory Factor Are Associated With Severe Malaria. Acta Tropica 89, 319–327. doi: 10.1016/j.actatropica.2003.10.010 14744558

[B26] Combes V. Coltel N. Alibert M. Van Eck M. Raymond C. Juhan-Vague I.. (2005). ABCA1 Gene Deletion Protects Against Cerebral Malaria. Am. J. Pathol. 166, 295–302. doi: 10.1016/S0002-9440(10)62253-5 15632021 PMC1602289

[B27] Combes V. Taylor T. E. Juhan-Vague I. Mège J.-L. Mwenechanya J. Tembo M.. (2004). Circulating Endothelial Microparticles in Malawian Children With Severe Falciparum Malaria Complicated With ComaRESEARCH LETTERS. JAMA: J. Am. Med. Assoc. 291, 2542–2544. doi: 10.1001/jama.291.21.2542-b 15173142

[B28] Daneman R. Prat A. (2015). The Blood–Brain Barrier. Cold Spring Harbor Perspect. Biol. 7, a020412. doi: 10.1101/cshperspect.a020412 PMC4292164 25561720

[B29] Datta D. Conroy A. L. Castelluccio P. F. Ssenkusu J. M. Park G. S. Opoka R. O.. (2020). Elevated Cerebrospinal Fluid Tau Protein Concentrations on Admission Are Associated With Long-Term Neurologic and Cognitive Impairment in Ugandan Children With Cerebral Malaria. Clin. Infect. Dis. 70, 1161–1168. doi: 10.1093/cid/ciz325 31044219 PMC7319060

[B30] Deininger M. H. Kremsner P. G. Meyermann R. Schluesener H. J. (2000). Differential Cellular Accumulation of Transforming Growth Factor–β1, –β2, and –β3 in Brains of Patients Who Died With Cerebral Malaria. J. Infect. Dis. 181, 2111–2115. doi: 10.1086/315493 10837206

[B31] Delahaye N. F. Coltel N. Puthier D. Barbier M. Benech P. Joly F.. (2007). Gene Expression Analysis Reveals Early Changes in Several Molecular Pathways in Cerebral Malaria-Susceptible Mice Versus Cerebral Malaria-Resistant Mice. BMC Genomics 8, 452. doi: 10.1186/1471-2164-8-452 18062806 PMC2246131

[B32] DellaValle B. Hempel C. Kurtzhals J. A. Penkowa M. (2010). In Vivo Expression of Neuroglobin in Reactive Astrocytes During Neuropathology in Murine Models of Traumatic Brain Injury, Cerebral Malaria, and Autoimmune Encephalitis. Glia 58, 1220–1227. doi: 10.1002/glia.21002 20544857

[B33] Diniz L. P. Almeida J. C. Tortelli V. Vargas Lopes C. Setti-Perdigão P. Stipursky J.. (2012). Astrocyte-Induced Synaptogenesis Is Mediated by Transforming Growth Factor β Signaling Through Modulation of D-Serine Levels in Cerebral Cortex Neurons. J. Biol. Chem. 287, 41432–41445. doi: 10.1074/jbc.M112.380824 23055518 PMC3510841

[B34] Dorovini-Zis K. Schmidt K. Huynh H. Fu W. Whitten R. O. Milner D.. (2011). The Neuropathology of Fatal Cerebral Malaria in Malawian Children. Am. J. Pathol. 178, 2146–2158. doi: 10.1016/j.ajpath.2011.01.016 21514429 PMC3081150

[B35] Dossi E. Vasile F. Rouach N. (2018). Human Astrocytes in the Diseased Brain. Brain Res. Bull. 136, 139–156. doi: 10.1016/j.brainresbull.2017.02.001 28212850 PMC5766741

[B36] Dvorak J. M. L. Whitehouse W. Shiroishi T. (1975). Invasion of Erythrocytes by Malaria Merozoites. Science 187, 748–750. doi: 10.1126/science.803712 803712

[B37] Endo F. Komine O. Fujimori-Tonou N. Katsuno M. Jin S. Watanabe S.. (2015). Astrocyte-Derived TGF-β1 Accelerates Disease Progression in ALS Mice by Interfering With the Neuroprotective Functions of Microglia and T Cells. Cell Rep. 11, 592–604. doi: 10.1016/j.celrep.2015.03.053 25892237

[B38] Eugenin E. A. Martiney J. A. Berman J. W. (2019). The Malaria Toxin Hemozoin Induces Apoptosis in Human Neurons and Astrocytes: Potential Role in the Pathogenesis of Cerebral Malaria. Brain Res. 1720, 146317. doi: 10.1016/j.brainres.2019.146317 31276637 PMC6702100

[B39] Fakhoury M. (2018). Microglia and Astrocytes in Alzheimer's Disease: Implications for Therapy. Curr. Neuropharmacol. 16, 508–518. doi: 10.2174/1570159X15666170720095240 28730967 PMC5997862

[B40] Figarella K. Wolburg H. Garaschuk O. Duszenko M. (2020). Microglia in Neuropathology Caused by Protozoan Parasites. Biol. Rev. 95, 333–349. doi: 10.1111/brv.12566 31682077

[B41] Gazzolo D. Michetti F. Bruschettini M. Marchese N. Lituania M. Mangraviti S.. (2003). Pediatric Concentrations of S100B Protein in Blood: Age- and Sex-Related Changes. Clin. Chem. 49, 967–970. doi: 10.1373/49.6.967 12765999

[B42] Grau G. E. MacKenzie C. D. Carr R. A. Redard M. Pizzolato G. Allasia C.. (2003). Platelet Accumulation in Brain Microvessels in Fatal Pediatric Cerebral Malaria. J. Infect. Dis. 187, 461–466. doi: 10.1086/367960 12552430

[B43] Hanisch B. R. Bangirana P. Opoka R. O. Park G. S. John C. C. (2015). Thrombocytopenia May Mediate Disease Severity in Plasmodium falciparum Malaria Through Reduced Transforming Growth Factor Beta-1 Regulation of Proinflammatory and Anti-inflammatory Cytokines. Pediatr. Infect. Dis. J. 34, 783–8. doi: 10.1097/INF.0000000000000729 PMC4466060 25886788

[B44] Hawkes M. Elphinstone R. E. Conroy A. L. Kain K. C. (2013). Contrasting Pediatric and Adult Cerebral Malaria: The Role of the Endothelial Barrier. Virulence 4, 543–555. doi: 10.4161/viru.25949 23924893 PMC5359751

[B45] Huang Y. Li S.-N. Zhou X.-Y. Zhang L.-X. Chen G.-X. Wang T.-H.. (2019). The Dual Role of AQP4 in Cytotoxic and Vasogenic Edema Following Spinal Cord Contusion and Its Possible Association With Energy Metabolism via COX5A. Front. Neurosci. 13, 584. doi: 10.3389/fnins.2019.00584 31258460 PMC6587679

[B46] Hu J. Ferreira A. Van Eldik L. J. (1997). S100β Induces Neuronal Cell Death Through Nitric Oxide Release From Astrocytes. J. Neurochem. 69, 2294–2301. doi: 10.1046/j.1471-4159.1997.69062294.x 9375660

[B47] Idro R. Jenkins N. E. Newton C. R. J. C. (2005). Pathogenesis, Clinical Features, and Neurological Outcome of Cerebral Malaria. Lancet Neurol. 4, 827–840. doi: 10.1016/S1474-4422(05)70247-7 16297841

[B48] Idro R. Kakooza-Mwesige A. Asea B. Ssebyala K. Bangirana P. Opoka R. O.. (2016). Cerebral Malaria Is Associated With Long-Term Mental Health Disorders: A Cross Sectional Survey of a Long-Term Cohort. Malaria J. 15, 184. doi: 10.1186/s12936-016-1233-6 PMC4815157 27030124

[B49] Idro R. Marsh K. John C. C. Newton C. R. (2010). Cerebral Malaria; Mechanisms Of Brain Injury And Strategies For Improved Neuro-Cognitive Outcome. Pediatr. Res. 68, 267. doi: 10.1203/PDR.0b013e3181eee738 20606600 PMC3056312

[B50] Ingebrigtsen T. Romner B. Kongstad P. Langbakk B. (1995). Increase Serum Concentration of Protein S-100 After Minor Head Injury: A Biochemical Serum Marker With Prognostic Value? J. Neurol. Neurosurg. Psychiatry 59, 103–104. doi: 10.1136/jnnp.59.1.103-a PMC1073618 7608699

[B51] Janota I. Doshi B. (1979). Cerebral Malaria in the United Kingdom. J. Clin. Pathol. 32, 769–777. doi: 10.1136/jcp.32.8.769 389955 PMC1145806

[B52] Jiang B.-C. He L.-N. Wu X.-B. Shi H. Zhang W.-W. Zhang Z.-J.. (2017). Promoted Interaction of C/Ebpα With Demethylated Cxcr3 Gene Promoter Contributes to Neuropathic Pain in Mice. J. Neurosci. 37, 685–700. doi: 10.1523/JNEUROSCI.2262-16.2016 28100749 PMC6596757

[B53] Lau K.Y. C. Turner L. Jespersen S. J. Lowe D. E. Petersen B. Wang W. C.. (2015). Structural Conservation Despite Huge Sequence Diversity Allows EPCR Binding by the PfEMP1 Family Implicated in Severe Childhood Malaria. Cell Host Microbe 17, 118–129. doi: 10.1016/j.chom.2014.11.007 25482433 PMC4297295

[B54] Liddelow S. Guttenplan K. Clarke L. Bennett F. Bohlen C. Schirmer L.. (2017). Neurotoxic Reactive Astrocytes Are Induced by Activated Microglia. Nature 541, 481. doi: 10.1038/nature21029 28099414 PMC5404890

[B55] Liu L. Li Y. Van Eldik L. J. Griffin W. S. T. Barger S. W. (2005). S100B-Induced Microglial and Neuronal IL-1 Expression Is Mediated by Cell Type-Specific Transcription Factors. J. Neurochem. 92, 546–553. doi: 10.1111/j.1471-4159.2004.02909.x 15659225

[B56] Lopez-Ramirez M. A. Wu D. Pryce G. Simpson J. E. Reijerkerk A. King-Robson J.. (2014). MicroRNA-155 Negatively Affects Blood–Brain Barrier Function During Neuroinflammation. FASEB J. 28, 2551–2565. doi: 10.1096/fj.13-248880 24604078

[B57] Lyke K. E. Sangare L. Diallo D. A. Taylor T. E. Cissoko Y. Doumbo O. K.. (2003). Association of Intraleukocytic Plasmodium Falciparum Malaria Pigment With Disease Severity, Clinical Manifestations, and Prognosis in Severe Malaria. Am. J. Trop. Med. Hyg. 69, 253–259. doi: 10.4269/ajtmh.2003.69.253 14628940

[B58] MacPherson G. G. Warrell M. J. White N. J. Looareesuwan S. Warrell D. A. (1985). Human Cerebral Malaria. A Quantitative Ultrastructural Analysis of Parasitized Erythrocyte Sequestration. Am. J. Pathol. 119, 385–401. 3893148 PMC1888001

[B59] Matejuk A. Ransohoff R. M. (2020). Crosstalk Between Astrocytes and Microglia: An Overview. Front. Immunol. 11, 1416. doi: 10.3389/fimmu.2020.01416 32765501 PMC7378357

[B60] Mathiisen T. M. Lehre K. P. Danbolt N. C. Ottersen O. P. (2010). The Perivascular Astroglial Sheath Provides a Complete Covering of the Brain Microvessels: An Electron Microscopic 3D Reconstruction. Glia 58, 1094–1103. doi: 10.1002/glia.20990 20468051

[B61] Matsuoka H. Yoshida S. Hirai M. Ishii A. (2002). A Rodent Malaria, Plasmodium Berghei, Is Experimentally Transmitted to Mice by Merely Probing of Infective Mosquito, Anopheles Stephensi. Parasitol. Int. 51, 17–23. doi: 10.1016/S1383-5769(01)00095-2 11880224

[B62] Mbagwu S. I. Lannes N. Walch M. Filgueira L. Mantel P.-Y. (2020). Human Microglia Respond to Malaria-Induced Extracellular Vesicles. Pathogens 9, 21. doi: 10.3390/pathogens9010021 PMC7168629 31878288

[B63] Medana I. M. Chan-Ling T. Hunt N. H. (1996). Redistribution and Degeneration of Retinal Astrocytes in Experimental Murine Cerebral Malaria: Relationship to Disruption of the Blood-Retinal Barrier. Glia 16, 51–64. doi: 10.1002/(SICI)1098-1136(199601)16:1<51::AID-GLIA6>3.0.CO;2-E 8787773

[B64] Medana I. M. Chaudhri G. Chan-Ling T. Hunt N. H. (2001). Central Nervous System in Cerebral Malaria: 'Innocent Bystander' or Active Participant in the Induction of Immunopathology? Immunol. Cell Biol. 79, 101–120. doi: 10.1046/j.1440-1711.2001.00995.x 11264703

[B65] Medana I. M. Day N. P. Hien T. T. Mai N. T. H. Bethell D. Phu N. H.. (2002). Axonal Injury in Cerebral Malaria. Am. J. Pathol. 160, 655–666. doi: 10.1016/S0002-9440(10)64885-7 11839586 PMC1850649

[B66] Medana I. M. Day N. P. Sachanonta N. Mai N. T. Dondorp A. M. Pongponratn E.. (2011). Coma in Fatal Adult Human Malaria Is Not Caused by Cerebral Oedema. Malaria J. 10, 267. doi: 10.1186/1475-2875-10-267 PMC3182981 21923924

[B67] Medana I. M. Hunt N. H. Chan-Ling T. (1997). Early Activation of Microglia in the Pathogenesis of Fatal Murine Cerebral Malaria. Glia 19, 91–103. doi: 10.1002/(SICI)1098-1136(199702)19:2<91::AID-GLIA1>3.0.CO;2-C 9034826

[B68] Medana I. M. Idro R. Newton C. R. J. C. (2007). Axonal and Astrocyte Injury Markers in the Cerebrospinal Fluid of Kenyan Children With Severe Malaria. J. Neurol. Sci. 258, 93–98. doi: 10.1016/j.jns.2007.03.005 17459417

[B69] Mohanty S. Benjamin L. A. Majhi M. Panda P. Kampondeni S. Sahu P. K.. (2017). Magnetic Resonance Imaging of Cerebral Malaria Patients Reveals Distinct Pathogenetic Processes in Different Parts of the Brain. mSphere 2, e00193–17. doi: 10.1128/mSphere.00193-17 PMC5463026 28596990

[B70] Pankoui Mfonkeu J. B. Gouado I. Fotso Kuaté H. Zambou O. Amvam Zollo P. H. Grau G. E. R.. (2010). Elevated Cell-Specific Microparticles Are a Biological Marker for Cerebral Dysfunctions in Human Severe Malaria. PloS One 5, e13415. doi: 10.1371/journal.pone.0013415 20976232 PMC2954805

[B71] Ponsford M. J. Medana I. M. Prapansilp P. Hien T. T. Lee S. J. Dondorp A. M.. (2012). Sequestration and Microvascular Congestion Are Associated With Coma in Human Cerebral Malaria. J. Infect. Dis. 205, 663–671. doi: 10.1093/infdis/jir812 22207648 PMC3266137

[B72] Promeneur D. Lunde L. K. Amiry-Moghaddam M. Agre P. (2013). Protective Role of Brain Water Channel AQP4 in Murine Cerebral Malaria. Proc. Natl. Acad. Sci. U. S. A. 110, 1035–1040. doi: 10.1073/pnas.1220566110 23277579 PMC3549120

[B73] Riggle B. A. Manglani M. Maric D. Johnson K. R. Lee M.-H. Neto O. L. A.. (2020). CD8+ T Cells Target Cerebrovasculature in Children With Cerebral Malaria. J. Clin. Invest. 130, 1128–1138. doi: 10.1172/JCI133474 31821175 PMC7269583

[B74] Schluesener H. Kremsner P. Meyermann R. (1998). Widespread Expression of MRP8 and MRP14 in Human Cerebral Malaria by Microglia Cells. Acta Neuropathol. 96, 575–580. doi: 10.1007/s004010050938 9845287

[B75] Schluesener H. J. Kremsner P. G. Meyermann R. (2001). Heme Oxygenase-1 in Lesions of Human Cerebral Malaria. Acta Neuropathol. 101, 65–68. doi: 10.1007/s004010000250 11194943

[B76] Shrivastava S. K. Dalko E. Delcroix-Genete D. Herbert F. Cazenave P. A. Pied S. (2017). Uptake of Parasite-Derived Vesicles by Astrocytes and Microglial Phagocytosis of Infected Erythrocytes May Drive Neuroinflammation in Cerebral Malaria. Glia 65, 75–92. doi: 10.1002/glia.23075 27696532

[B77] Steiner H. Fukumori A. Tagami S. Okochi M. (2018). Making the Final Cut: Pathogenic Amyloid-Beta Peptide Generation by Gamma-Secretase. Cell Stress 2, 292–310. doi: 10.15698/cst2018.11.162 31225454 PMC6551803

[B78] Susomboon P. Maneerat Y. Dekumyoy P. Kalambaheti T. Iwagami M. Komaki-Yasuda K.. (2006). Down-Regulation of Tight Junction mRNAs in Human Endothelial Cells Co-Cultured With Plasmodium Falciparum-Infected Erythrocytes. Parasitol. Int. 55, 107–112. doi: 10.1016/j.parint.2005.11.054 16388977

[B79] Talavera-López C. Capuccini B. Mitter R. Lin J.-W. Langhorne J. (2018). Transcriptomes of Microglia in Experimental Cerebral Malaria in Mice in the Presence and Absence of Type I Interferon Signaling. BMC Res. Notes 11, 913. doi: 10.1186/s13104-018-4020-3 30572937 PMC6302474

[B80] Taramelli D. Basilico N. Pagani E. Grande R. Monti D. Ghione M.. (1995). The Heme Moiety of Malaria Pigment (β-Hematin) Mediates the Inhibition of Nitric Oxide and Tumor Necrosis Factor-α Production by Lipopolysaccharide-Stimulated Macrophages. Exp. Parasitol. 81, 501–511. doi: 10.1006/expr.1995.1143 8542991

[B81] Taramelli D. Recalcati S. Basilico N. Olliaro P. Cairo G. (2000). Macrophage Preconditioning With Synthetic Malaria Pigment Reduces Cytokine Production via Heme Iron-Dependent Oxidative Stress. Lab. Invest. 80, 1781–1788. doi: 10.1038/labinvest.3780189 11140691

[B82] Taylor R. A. Chang C.-F. Goods B. A. Hammond M. D. Grory B. M. Ai Y.. (2016). TGF-β1 Modulates Microglial Phenotype and Promotes Recovery After Intracerebral Hemorrhage. J. Clin. Invest. 127, 280–292. doi: 10.1172/JCI88647 27893460 PMC5199690

[B83] Thelin E. P. Zibung E. Riddez L. Nordenvall C. (2016). Assessing Bicycle-Related Trauma Using the Biomarker S100B Reveals a Correlation With Total Injury Severity. Eur. J. Trauma Emerg. Surg. 42, 617–625. doi: 10.1007/s00068-015-0583-z 26490563

[B84] Thurgur H. Pinteaux E. (2018). Microglia in the Neurovascular Unit: Blood-Brain Barrier-microglia Interactions After Central Nervous System Disorders. Neuroscience 405, 55–67. doi: 10.1016/j.neuroscience.2018.06.046 31007172

[B85] Tripathi A. K. Sullivan D. J. Stins M. F. (2006). Plasmodium Falciparum-Infected Erythrocytes Increase Intercellular Adhesion Molecule 1 Expression on Brain Endothelium Through NF-KappaB. Infect. Immun. 74, 3262–3270. doi: 10.1128/IAI.01625-05 16714553 PMC1479273

[B86] Tripathi A. K. Sullivan D. J. Stins M. F. (2007). Plasmodium Falciparum–Infected Erythrocytes Decrease the Integrity of Human Blood-Brain Barrier Endothelial Cell Monolayers. J. Infect. Dis. 195, 942–950. doi: 10.1086/512083 17330783

[B87] Turner G. D. Morrison H. Jones M. Davis T. M. Looareesuwan S. Buley I. D.. (1994). An Immunohistochemical Study of the Pathology of Fatal Malaria. Evidence for Widespread Endothelial Activation and a Potential Role for Intercellular Adhesion Molecule-1 in Cerebral Sequestration. Am. J. Pathol. 145, 1057–1069. 7526692 PMC1887431

[B88] Velagapudi R. Kosoko A. M. Olajide O. A. (2019). Induction of Neuroinflammation and Neurotoxicity by Synthetic Hemozoin. Cell. Mol. Neurobiol. 39, 1187–1200. doi: 10.1007/s10571-019-00713-4 31332667 PMC6764936

[B89] Vilhardt F. Haslund-Vinding J. Jaquet V. Mcbean G. (2017). Microglia Antioxidant Systems and Redox Signalling. Br. J. Pharmacol. 174, 1719–1732. doi: 10.1111/bph.13426 26754582 PMC5446583

[B90] Weiss G. Gilson P. R. Taechalertpaisarn T. Tham W. H. De Jong N. W. Harvey K. L.. (2015). Revealing the Sequence and Resulting Cellular Morphology of Receptor-Ligand Interactions During Plasmodium Falciparum Invasion of Erythrocytes. PloS Pathog. 11, e1004670. doi: 10.1371/journal.ppat.1004670 25723550 PMC4344246

[B91] White N. J. (2008). Plasmodium Knowlesi: The Fifth Human Malaria Parasite. Clin. Infect. Dis. an Off. Publ. Infect. Dis. Soc. America 46, 172–173. doi: 10.1086/524889 18171246

[B92] WHO. (2014). “Severe Malaria Section 1: Epidemiology of Severe Falciparum Malaria,” in Tropical Medicine and International Health (John Wiley & Sons), 19(s1), 7–131. doi: 10.1111/tmi.12313 25214480

[B93] Wilson K. D. Ochoa L. F. Solomon O. D. Pal R. Cardona S. M. Carpio V. H.. (2018). Elimination of Intravascular Thrombi Prevents Early Mortality and Reduces Gliosis in Hyper-Inflammatory Experimental Cerebral Malaria. J. Neuroinflamm. 15, 173. doi: 10.1186/s12974-018-1207-4 PMC5987620 29866139

[B94] Woertgen C. Rothoerl R. D. Brawanski A. (2002). Early S-100B Serum Level Correlates to Quality of Life in Patients After Severe Head Injury. Brain Inj 16, 807–816. doi: 10.1080/02699050210128933 12217206

[B95] World Health Organization. (2020). World Malaria Report 2020: 20 Years of Global Progress and Challenges (Geneva, Switizerland). Available at: https://apps.who.int/iris/handle/10665/337660.

[B96] Xia M. Q. Bacskai B. J. Knowles R. B. Qin S. X. Hyman B. T. (2000). Expression of the Chemokine Receptor CXCR3 on Neurons and the Elevated Expression of Its Ligand IP-10 in Reactive Astrocytes: In Vitro ERK1/2 Activation and Role in Alzheimer’s Disease. J. Neuroimmunol. 108, 227–235. doi: 10.1016/S0165-5728(00)00285-X 10900358

[B97] Zhang Z. Ma Z. Zou W. Guo H. Liu M. Ma Y.. (2019). The Appropriate Marker for Astrocytes: Comparing the Distribution and Expression of Three Astrocytic Markers in Different Mouse Cerebral Regions. BioMed. Res. Int. 2019, 1–15. doi: 10.1155/2019/9605265 PMC6613026 31341912

